# Safety and efficacy of direct cardiac shockwave therapy in patients with ischemic cardiomyopathy undergoing coronary artery bypass grafting (the CAST-HF trial): study protocol for a randomized controlled trial—an update

**DOI:** 10.1186/s13063-022-06931-4

**Published:** 2022-12-09

**Authors:** Felix Nägele, Leo Pölzl, Michael Graber, Jakob Hirsch, Agnes Mayr, Mathias Pamminger, Felix Troger, Markus Theurl, Michael Schreinlechner, Nikolay Sappler, Christian Dorfmüller, Martina Mitrovic, Hanno Ulmer, Michael Grimm, Can Gollmann-Tepeköylü, Johannes Holfeld

**Affiliations:** 1 Department of Cardiac Surgery, Medical University of Innsbruck, Innsbruck, Austria; 2 Department of Radiology, Medical University of Innsbruck, Innsbruck, Austria; 3 Department of Internal Medicine III, Medical University of Innsbruck, Innsbruck, Austria; 4 Heart Regeneration Technologies, Innsbruck, Austria; 5 Department of Medical Statistics, Informatics and Health Economics, Innsbruck, Austria

**Keywords:** Shockwave, CABG, Ischemic heart disease, Heart failure, Clinical trial

## Abstract

**Background:**

Coronary artery disease (CAD) remains a severe socio-economic burden in the Western world. Coronary obstruction and subsequent myocardial ischemia result in the progressive replacement of contractile myocardium with dysfunctional, fibrotic scar tissue. Post-infarctional remodelling is causal for the concomitant decline of left-ventricular function and the fatal syndrome of heart failure. Available neurohumoral treatment strategies aim at the improvement of symptoms. Despite extensive research, therapeutic options for myocardial regeneration, including (stem)-cell therapy, gene therapy, cellular reprogramming or tissue engineering, remain purely experimental. Thus, there is an urgent clinical need for novel treatment options for inducing myocardial regeneration and improving left-ventricular function in ischemic cardiomyopathy. Shockwave therapy (SWT) is a well-established regenerative tool that is effective for the treatment of chronic tendonitis, long-bone non-union and wound-healing disorders. In preclinical trials, SWT regenerated ischemic myocardium via the induction of angiogenesis and the reduction of fibrotic scar tissue, resulting in improved left-ventricular function.

**Methods:**

In this prospective, randomized controlled, single-blind, monocentric study, 80 patients with reduced left-ventricular ejection fraction (LVEF≤ 40%) are subjected to coronary-artery bypass-graft surgery (CABG) surgery and randomized in a 1:1 ratio to receive additional cardiac SWT (intervention group; 40 patients) or CABG surgery with sham treatment (control group; 40 patients). This study aims to evaluate (1) the safety and (2) the efficacy of cardiac SWT as adjunctive treatment during CABG surgery for the regeneration of ischemic myocardium. The primary endpoints of the study represent (1) major cardiac events and (2) changes in left-ventricular function 12 months after treatment. Secondary endpoints include 6-min walk test distance, improvement of symptoms and assessment of quality of life.

**Discussion:**

This study aims to investigate the safety and efficacy of cardiac SWT during CABG surgery for myocardial regeneration. The induction of angiogenesis, decrease of fibrotic scar tissue formation and, thus, improvement of left-ventricular function could lead to improved quality of life and prognosis for patients with ischemic heart failure. Thus, it could become the first clinically available treatment strategy for the regeneration of ischemic myocardium alleviating the socio-economic burden of heart failure.

**Trial registration:**

ClinicalTrials.gov NCT03859466. Registered on 1 March 2019.

## Introduction

### Background and rationale {6a}

Myocardial viability dictates survival and functional improvement after coronary artery bypass grafting (CABG) in patients with postischemic congestive heart failure [1, 2]. The success of surgical revascularisation is limited by non-viable myocardium. Therefore, adjunctive therapies inducing myocardial regeneration, applied during surgery, could improve survival and functional outcome in patients with postischemic congestive heart failure. To gain broad clinical applicability, adjunctive myocardial regenerative therapies must be efficient, easily applicable and safe.

Various cell lines, including autologous myoblasts and stem cells, induce myocardial regeneration when applied in adjunction to coronary artery bypass grafting [3, 4]. However, the production of cells is complex, carries a significant financial burden and can be accompanied by serious adverse events [5, 6].

Shock wave therapy has been used to treat kidney stones for the past 20 years [7]. If applied at low energy levels, shock waves can regenerate infarcted and chronic ischemic myocardium. Shock waves can be delivered percutaneously or direct epicardially. Percutaneous cardiac shock wave therapy improves clinical symptoms in patients with chronic ischemic myocardium. However, percutaneous cardiac shock wave therapy is limited by appropriate acoustic windows — shock waves cannot safely be applied over lung tissue [8, 9]. Therefore, only selected areas of the myocardium — primarily the anterior wall — can be treated by percutaneous cardiac shock wave therapy. Direct epicardial shock wave therapy (DESWT) may develop as an adjunctive therapy to CABG, especially in those patients with large myocardial infarcts or areas of non-reversible ischemia. Up to date, no published data on DESWT exist [10].

The objective of the CAST-HF trial, therefore, is to evaluate the effect of direct epicardial shockwave therapy adjunctive to CABG surgery in patients suffering from markedly reduced ejection fraction. For this study, a newly developed device specifically designed for direct cardiac treatment will be tested.

### Objectives {7}

Primary:

- To assess the safety profile of the device
- To assess the Improvement of left ventricular ejection fraction (LVEF) by using cardiac shock wave therapy in patients undergoing primary coronary artery bypass grafting and suffering from reduced left ventricular function below or equal 40%

Secondary:

- To assess the long-term safety profile of the device
- To assess the long-term performance profile of the device

Exploratory:

- To evaluate gene expression profiles associated with cardiac shockwave therapy

### Trial design {8}

SPIRIT guidance: Description of trial design including type of trial (e.g. parallel-group, crossover, factorial, single group), allocation ratio, and framework (e.g. superiority, equivalence, noninferiority, exploratory).

The CAST-HF trial is a randomized, single-blind, parallel-group, sham-controlled superiority trial.

## Methods: Participants, interventions and outcomes

### Study setting {9}

All participants will be recruited by the medical specialist team supported by a study nurse (recruiting team) at the Department of Cardiac Surgery, Medical University Innsbruck (MUI) (Investigation Site). A data safety monitoring board (DSMB) consisting of external experts is established to review patient’s data for inclusion, exclusion or early study termination. All data will be collected by the research team consisting of physicians and healthcare researchers. Data analysis will be performed by the Department of Medical Statistics, Informatics, and Health Economics, Innsbruck Medical University, Innsbruck, Austria.

### Eligibility criteria {10}

Adult patients with postischemic heart failure undergoing coronary artery bypass grafting (CABG). All participants will undergo the usual standard medical work-up before, during, and after CABG according to the ESC/EACTS Guidelines on Myocardial Revascularization. This includes Coronary Angiography, Echocardiography, Spirometry, Carotid Artery Duplex Scan, Cardiac MRI, Computed Tomography, and interviews with the Cardiologist, Cardiac Surgeon, and Anesthesiologist.

Inclusion criteria:

- Male or female patients above 21 and under 90 years of age undergoing primary coronary artery bypass grafting.
- Patients have to present with reduced left ventricular function defined as LVEF ≤ 40%.
- Patients have to present with regional left ventricular wall motion abnormalities.
- Patients have to give written informed consent to participate in the study.

Exclusion criteria

- Significant concomitant aortic valve disease in need of surgical treatment (Except significant aortic valve disease not detected in preoperative cardiac ultrasound that is detected intra-operatively).
- Serious radiographic contrast allergy.
- Patients in cardiogenic shock or presenting with acute myocardial infarction (STEMI or NSTEMI)
- Patients with a contraindication for cardiac MRI (e.g. glomerular filtration rate <30ml/min/1.73m^2^)
- History of significant ventricular arrhythmias, except arrhythmias associated with MI.
- Present co-morbidity, which reduces life expectancy to less than 1 year.
- Presence of ventricular thrombus.
- Presence of a cardiac tumour.
- Pregnancy.

### Who will take informed consent? {26a}

Every patient must give his/her written consent before participating in the clinical trial. Before the patient gives his/her written consent, the patient has to be informed completely in oral and written form in an understandable manner about the patient about character, importance, relevance and consequences of the clinical trial by one of the study investigators whom the principal investigator specifically delegates.

The content of the consent information has to be documented on the Informed Consent. The patient will be notified if essential findings about the MD appear during the study.

The Informed Consent of the patient about participation in the clinical trial must be dated and signed by the patient and the study investigator. The patient receives one exemplar of the signed and dated Patient Information and Informed Consent Form. The investigator must store the second signed and dated exemplar in the Investigator Site File.

It must be explicitly pointed out that until valid Informed Consent of the patient is existent no study-specific investigations carried out with the patient are allowed.

### Additional consent provisions for collection and use of participant data and biological specimens {26b}

The following additional collections and use of participant data and biological specimens are explicitly stated in the informed consent file: left-ventricular biopsies, biosamples. Details on collection of these samples are described in section 33. Additionally, patients are informed in oral form and in an understandable manner about the character, importance, relevance and consequences of these collections.

## Interventions

### Explanation for the choice of comparators {6b}

The control group receives CABG surgery without DESWT, which is the state-of-the-art technique for the treatment of myocardial infarction due to coronary artery disease. Instead of DESWT, a Sham Treatment will be performed. For this reason, a non-functional applicator will be held on the exact same areas of the heart for the same amount of time, as the in patients of the treatment group. Thus, the control patients receive the same manipulation of the specific areas as the patients in the treatment arm.

### Intervention description {11a}

Both the experimental intervention and the sham procedure are performed during open, on-pump CABG surgery indicated by the institutional heart team of interventional cardiologists and cardiac surgeons.

#### Experimental (intervention) arm

In the intervention arm, 300 shockwave impulses per coronary supply territory, at an energy flux density of 0.38mJ/mm2 and a frequency 3Hz, are applied in direct contact with the ischaemic myocardium of the left ventricle. The intervention is performed during CABG surgery after bypasses are fully established while still on cardiopulmonary bypass by the same cardiac surgeon performing the CABG procedure whom the principal investigator delegates. No specific surgical skills are needed for the application of DESWT with the Cardiac Shockwave Probe; however, investigators performing the procedure are trained and supervised by both the principal investigator and technicians to ensure reproducibility.

The cardiac shockwave system consists of a table-top device (Nonvasiv Medical GmbH, Konstanz, Germany) and a sterile single-use applicator releasing electrohydraulic shockwaves (Heart Regeneration Technologies GmbH, Innsbruck, Austria). Prior to use, the applicator is inserted into a sterile cover containing ultrasound gel. In order to ensure acoustic coupling between the applicator and the myocardium, continuous saline rinsing is applied throughout the procedure.

#### No intervention (sham control) arm

In the sham control arm, the same manipulations are performed with an inactive shockwave applicator in direct contact with the ischaemic myocardium of the left ventricle as in the intervention arm. The sham treatment is performed during CABG surgery after bypasses are fully established while still on cardiopulmonary bypass.

### Criteria for discontinuing or modifying allocated interventions {11b}

Subjects will exit the clinical investigation for any of the following reasons:

- Screening failure
- Withdrawal of informed consent
- Lost to follow-up
- Death

The subject is free to withdraw its written informed consent at any time. The reason for withdrawal should, if available, be documented in the subject’s medical file and in the CRF. When a subject withdraws or is withdrawn from the clinical investigation, a final clinical evaluation should be performed, documented in the medical record and in the CRF.

A subject is not considered lost to follow-up until the full study period has elapsed. The investigator should try to contact the subjects at every follow-up until clinical investigation ends.

### Strategies to improve adherence to interventions {11c}

To improve adherence to the study protocol, the follow-up visits for the study are planned simultaneously with the standard clinical follow-up appointments for patients in the high-risk cardiac surgery program at the Department for Cardiac Surgery, Medical University of Innsbruck. Apart from showing up for the follow-up appointments, participants do not need to adhere to specific tasks.

### Relevant concomitant care permitted or prohibited during the trial {11d}

There is no expected interaction between the device under investigation and concomitant medical treatments.

Throughout the study, investigators are permitted to use their clinical judgement when prescribing concomitant medications and treatments for trial patients. Local prescribing information and institutional guidelines should be followed as applicable.

#### Optimized, guideline-directed medical therapy

This study is designed to be an all-comers study. Optimized medical therapy according to heart failure guidelines of the European Society of Cardiology (PMID: 34447992) may already be achieved at the time of screening and treatment or not. In any case, at the time of follow-up visits to assess LVEF via cardiac MRI (D180, D360) patients have to be on stable, guideline-directed medical therapy for at least 30 days prior to each visit.

This includes the following definitions according to the patients’ clinical presentation and the judgement of the responsible physician:

- No more than a 100% increase or a 50% decrease of the dosage of any one medication other than a diuretic.
- Medication changes within a drug class are allowed as long as the equivalent dosage is within the limits specified above.
- Unrestricted changes in diuretics are allowed as long as the subject remains on a diuretic.

### Provisions for post-trial care {30}

The Sponsor will take out reasonable third-party liability insurance cover in accordance with local legal requirements. The civil liability of the Investigator, all persons instructed and the hospital, practice or institute in which they are employed and the liability of the Sponsor in respect of financial loss due to personal injury and other damage that may arise as a result of the carrying out of this study are governed by the applicable local law.

As a precautionary measure, the Investigator, the persons instructed and the hospital, practice or institute are included in such cover in terms of their work done in carrying out the study to the extent that the claims are not covered by their own professional indemnity insurance.

The Sponsor will arrange for patients participating in this study to be insured against financial loss due to personal injury caused by the device being tested or by medical steps taken in the course of the study. Such insurance is taken out by the Sponsor in accordance with or by way of analogy to both the Austrian and the other participating countries’ drug law.

### Outcomes {12}

Primary endpoints:

- Efficacy: Improvement in LVEF measured by cardiac MRI from baseline to 360 days.
- Safety: Occurrence of device-related complications (adverse device effects or serious adverse device effects) within 360 days.

Secondary endpoints:

- Endpoints related to the efficacy are changes in the following parameters within a time frame of 360 days:
  - 6-min walk test distance
  - NYHA functional class
  - Serum nt-proBNP levels
  - Renal function, measured by glomerular filtration rate (GFR)
  - Seattle Angina Pectoris Questionnaire (SAQ)
  - 36-item short-form survey (SF36)
  - Minnesota Living with Heart Failure Questionnaire (MLHFQ)
- Secondary safety-related endpoints include the following observations within a time frame of 6 days:
  - Occurrence of ventricular arrhythmia during the hospital stay
  - Occurrence of device-related peri-operative myocardial damage detected by a rise of cardiac biomarkers: creatine kinase (CK-MB) and troponin T (TropT)
  - Occurrence of signs of device-related infection detected by a rise of C-reactive protein (CRP) or leukocytosis during the hospital stay

### Participant timeline {13}

#### Sample size {14}

Based on prior findings from 1 pilot study (intervention effect, unpublished) and 1 meta-analysis (control effect) [11], two a priori sample size calculations (1 conservative and 1 liberal method) were executed for the primary performance outcome “LVEF” by means of G*power v 3.1.9.2 (University of Kiel, Germany). As effect sizes for multivariate analysis are not available till now (partial ƞ^2^), the sample size calculation was executed based on baseline/follow-up differences between groups (Cohen’s *d*). For both calculations, an alpha risk of 0.05 and a statistical power of 0.8 were tolerated.

#### Conservative method

This method is considered the highest standard deviation found in both aforementioned studies. Assuming an effect size (Cohens d) of 0.678 for baseline/follow-up differences between groups (mean improvement; intervention: 10.4 ± 8.7, control 4.5 ± 8.7) on LVEF, 72 participants (36 intervention/36 control) were needed for statistical analysis with an adequate power, assuming an alpha risk of 0.05 (two-sided) and a beta risk of 0.20 (non-normal distributed). With an estimated loss to follow-up (mortality and dropout) of 10%, 80 patients have to be enrolled to ensure that 72 patients can be analysed.

#### Liberal method

This method considered the lowest standard deviation for each group type (control vs. intervention) found in both aforementioned studies. Assuming an effect size (Cohen’s d) of 0.903 for the baseline/follow-up differences between groups (mean improvement; intervention: 10.4 ± 7.22, control 4.5 ± 5.76) on LVEF, 32 participants (16 intervention/16 control) were needed for statistical analysis with an adequate power, assuming an alpha risk of 0.05 (one-sided) and a beta risk of 0.20 (normal distributed). With an estimated loss to follow-up (mortality and dropout) of 10%, 36 patients have to be enrolled to ensure that 32 patients can be analysed.

To ensure sufficient statistical power, 80 participants (conservative method) will be implemented in this study. Nevertheless, due to ethical considerations (i.e. implement not more participants than necessary), an interim analysis will be executed after 20 vs. 20 participants have been taken into account (10% above the liberal method) in order to stop the recruiting process when sufficient statistical power has been reached.

### Recruitment {15}

Patients are recruited from University Hospital for Cardiac Surgery, Medical University of Innsbruck, Innsbruck, Austria, starting in November 2018. No specific measurements will be taken. Given the structure of the Austrian health care system, this department is the only institution in the allocated region providing cardiac surgery. All patients with a reduced LVEF admitted to CABG surgery, as decided by the institutional heart team, will be screened for eligibility by the study investigators. The approximate recruitment rate is expected to be 20 patients per year.

## Assignment of interventions: allocation

### Sequence generation {16a}

The investigational medicinal product (shockwave therapy) to which individual patients will be assigned is determined by a randomized schedule with a 1:1 allocation ratio. The sequence allocation will be generated in permuted blocks by an independent statistician. The Competence Centre for Clinical Trials at the Medical University of Innsbruck will have access to the allocation sequence to prepare the individual opaque envelopes for each participant.

### Concealment mechanism {16b}

The randomization process will be realized independently from the clinical investigators using opaque envelops (fully blind randomization) For each participant enrolled on the study, one envelope will be opened at the end of the CABG procedure after opening the aortic clamp (bypasses fully established) while the patient is still connected to the heart-lung machine to get group allocation information.

### Implementation {16c}

SPIRIT guidance: Who will generate the allocation sequence, who will enrol participants, and who will assign participants to interventions.

An independent statistician from an external company will provide the randomization list. The opaque envelopes will be prepared by the Clinical Trials Monitoring Center of Innsbruck Medical University.

A participant can be included by every member of the research team; when in doubt, the inclusion will be judged by the whole research team and reviewed by the clinical trial monitoring board anyways.

## Assignment of interventions: blinding

### Who will be blinded {17a}

This study is single-blinded as the PI, and the surgical personnel involved in the study procedure (shockwave therapy or sham treatment) know the randomization arm. However, all study assessments will be performed in an observer-blinded fashion. Patients, the investigators performing the endpoint analyses, and data analysts are blinded to the group assignment.

### Procedure for unblinding if needed {17b}

Unblinding is not applicable in this study as no continuous study intervention (e.g. medication) is used but a single intra-operative treatment. Documentation and processing of device-related adverse events do not require unblinding.

## Data collection and management

### Plans for assessment and collection of outcomes {18a}

#### Data collection and management

Baseline, outcomes and other trial data will be collected by the study investigators. Monitoring and audits will be performed for quality assurance within the clinical investigation. Monitoring and auditing procedures developed or endorsed by the Sponsor will be adhered to, in order to comply with EN ISO 14155 guidelines and local legal requirements to ensure the acceptability of the study data. Regulatory authorities, the ethics committees, and Sponsor’s delegates may perform on-site inspections or audits, for which the Investigator must provide support at all times. All collected data of this study have to be recorded in the CRF by appropriate authorized persons. This is also valid for data of patients, who dropped out of the study. Further details on data entry and handling are specified in section 19.

#### Study-specific training

All investigators will be trained within the Initiation Visit. Training topics cover Good Clinical Practice, the study protocol, and the operation of the DSP-002 Flashwave MMC and the Cardiac Shockwave Probe (CSP). The training will be documented on a specific training log and the respective delegations will be documented on the Delegation Log Form. No specific surgical skills are needed for the application of DESWT with the Cardiac Shockwave Probe. Details about the application are provided in section 11.

#### Medication

Throughout the study, investigators are permitted to use their clinical judgement when prescribing concomitant medications and treatments for trial patients. Local prescribing information and institutional guidelines should be followed as applicable.

#### Optimized, guideline-directed medical therapy

This study is designed to be an all-comers study. Optimized medical therapy according to the European Society of Cardiology Guidelines for the diagnosis and treatment of acute and chronic heart failure may already be achieved at the time of screening and treatment or not. At the time of follow-up visits to assess LVEF via cardiac MRI (D180, D360) patients have to be on stable, guideline-directed medical therapy for at least 30 days prior to each visit. This includes the following definitions according to the patients’ clinical presentation and the judgement of the responsible physician:

- No more than a 100% increase or a 50% decrease of the dosage of any one medication other than a diuretic.
- Medication changes within a drug class are allowed as long as the equivalent dosage is within the limits specified above.

#### Cardiac MRI

All CMR examinations will be performed on a 1.5-T clinical MR imaging unit (AVANTO_fit; Siemens, Erlangen, Germany) according to a multiparametric protocol including the following sequences:

- Cine-SSFP: Cine CMR images in short-axis slices covering LV cavity and in long-axis slices (2-, 3- and 4-chamber view) will be acquired using a breath hold, retrospective ECG-triggered steady-state free-precession (SSFP) trueFISP bright-blood sequence with the following typical imaging parameters: Voxel size = 1.5 × 1.5 × 6 mm^3^, echo time = 1.13 ms, time to repetition = 38.92 ms, flip angle = 80°, parallel imaging mode: generalized auto-calibrating partially parallel acquisition (GRAPPA), acceleration factor = 2.
- Late enhancement CMR images will be acquired by using an ECG-triggered phase-sensitive inversion recovery (PSIR) single-shot TrueFISP sequence with consecutive short-axis slices 10 min after an intravenous administration of 0.15 mmol/kg of Gd-DO3A-butriol (GadovistTM, Bayer Vital, Leverkusen, Germany) at 2 ml/s, followed by 20 ml of saline flush, administered using an automatic injector (Spectris Injection System, Medrad, Pittsburgh, USA) with the following typical imaging parameters: voxel size = 1.4 × 1.4 × 8 mm^3^, echo time = 1.22 ms, time to repetition = 945.60 ms, flip angle = 45°.
- Mapping sequences
  - T1 Mapping: A single breath-hold, ECG-gated short modified Look-Locker inversion recovery (shMOLLI) sequence will be acquired on a LV basal, mid-ventricular and apical short-axis slice at end-diastole before and 20 min after contrast media administration. Typical imaging parameters are voxel size = 1.4 × 1.4 × 8 mm^3^, four single shot balanced SSFP readout trains with effective inversion times between 424 and 1439.36 ms, echo time = 1.13 ms, time to repetition = 359.84 ms, flip angle = 35°, parallel imaging mode GRAPPA with an acquisition factor of 2, and slice thickness = 8 mm. A motion correction algorithm will be applied to allow for movement artefacts.
  - T2 Mapping: T2 quantification will be performed for characterization of myocardial oedema. Three matching short-axis T2-weighted images will be obtained before administration of contrast media using a free-breathing, ECG-gated, T2-prepared trueFISP sequence at 0, 25, and 55 ms, allowing generation of the T2 time for each individual voxel. Again, a motion correction algorithm will be applied to allow for movement artefacts. Typical imaging parameters are: echo time = 1.06 ms, repetition time = 187.37 ms, flip angle = 70°, bandwidth = 1184 Hz/pixel, matrix = 192 × 112 pixels, spatial resolution = 2.0 × 2.0 × 8.0 mm, and slice thickness = 8 mm.
  - T2* Mapping: Myocardial haemorrhage will be assessed by T2* quantification using a breath-hold, cardiac gated gradient echo sequence with 8 echoes obtained in three matching short-axis slices before administration of contrast media. Again, a motion correction algorithm will be applied to allow for movement artefact. Typical imaging parameters are echo time = 2.02–16.3 ms, time to repetition = 948.8 ms, flip angle = 20°, bandwidth = 814 Hz/pixel, matrix = 256 × 110 pixels, spatial resolution = 1.6 × 1.6 × 8.0 mm, and slice thickness = 8 mm.
- Postprocessing analysis
  - Cine-SSFP: Left ventricular (LV) volumes, LV myocardial volume and mass as well as LV ejection fraction (EF) will be analysed via standard software (Circle Cardiovascular Imaging, Calgary, Canada) for postprocessing analyses with semi-automatic detection of LV endo- and epicardial contours
  - Late enhancement: Infarct characteristics (infarct location, infarct size, infarct transmurality, microvascular obstruction) will be determined as myocardial areas with signal intensity above the average of apparently normal myocardium in the opposite noninfarcted myocardial segments plus 5 standard deviations. Planimetry of late enhancement areas will be performed on IMPAX EE workstation (Agfa Healthcare, Bonn, Germany).
  - Mapping sequences: Motion-corrected, colour-coded T1 native -, T1 post contrast -, T2 - and T2*- myomaps will be automatically inline generated by fitting signal intensities at each image pixel with an exponential model for the given echo times. Evaluation of the maps will be carried out on IMPAX EE workstations (Agfa Healthcare, Bonn, Germany) and performed by manually outlining endo- and epicardial contours with taking care to exclude subendocardial blood and subepicardial tissue to avoid partial volume effects.LV myocardium will subsequently be divided into regions of interest (ROI): infarct core, peri-infarct zone and remote myocardium, defined as the segment contralateral to the infarct segment, by drawing manual contours over the corresponding maps and visually inspecting viability on late enhancement images. Extracellular volume (ECV) values will be generated using a previously established equation. R1 will be defined as 1/T1 and delta will be defined as the difference between native and post-contrast T1 values. T2 maps (ECG-gated, T2 prepared trueFISP sequence at 0, 25, and 55 ms in matching slices before administration of contrast media) and T2* maps (ECG-gated gradient echo sequence with 8 echoes) will be obtained in matching slices before administration of contrast media.

#### Laboratory analysis

Samples of laboratory tests will be analysed at the local laboratory.

Blood analysis will be done as part of the regular safety assessments at baseline, at hospital discharge and at visits in the long-term follow-up.

The following parameters will be measured:

- Laboratory results general:
  - Lactatdehydrogenase
  - Erythrocytes
  - Haemoglobin
  - Haematocrit
  - Platelet count
  - CRP
  - Leukocytes
  - Thrombo test
  - Activated partial thromboplastin time
  - International normalized ratio
  - Pro BNP
- Laboratory results heart enzymes:
  - Total CK
  - CK-MB
  - CK-MB %
  - Troponin T

#### Echocardiography

Echocardiography has become routinely used in the diagnosis, management, and follow-up of patients with any suspected or known heart diseases. It is one of the most widely used diagnostic tests in cardiology. It can provide a wealth of helpful information, including the size and shape of the heart (internal chamber size quantification), pumping capacity, and the location and extent of any tissue damage. An echocardiogram can also give physicians other estimates of heart function, such as a calculation of the cardiac output, ejection fraction, and diastolic function. The echocardiography will be performed according to the routinely performed assessment protocol transthoracic, but during surgery transoesophageal.

#### Six-min walk test

The 6-min walk test is a sub-maximal exercise test used to assess aerobic capacity and endurance. The distance covered over a time of 6 min is used as the outcome by which to compare changes in performance capacity. The test will be performed indoors along a long, flat, straight, enclosed corridor with a hard surface without a warm-up period after a rest of 10 min, while the patient is sitting in a chair located near the starting position. Thereafter we have the patient stand and rate his/her baseline dyspnoea and overall fatigue using the Borg scale. The patient has to be instructed to walk as far as possible for 6 min without running of jogging. To measure time and walking distance, a timer and a lap counter are used. While walking the patient should be informed every minute how long he has to go. After the test, the postwalk dyspnoea and fatigue levels will be recorded. Blood pressure and heart rate will be measured at baseline and postwalk. Resting during the test is allowed, but time keeps running. The test will be stopped, if the patient develops chest pain, intolerable dyspnea, staggering, diaphoresis, intolerable cramps, and/or an ashen appearance.

#### SF36

SF-36 is a set of generic, coherent, and easily administered quality-of-life measures. These measures rely upon patient self-reporting and are now widely utilized by managed care organizations and by Medicare for routine monitoring and assessment of care outcomes in adult patients.

#### Seattle Angina Pectoris Questionnaire

The SAQ is a self-administered, disease-specific measure for reproducible, and sensitive to clinical change. This instrument was developed and validated by John Spertus, Director of Cardiovascular Education and Outcomes Research at the Mid America Heart Institute and Professor of Medicine at the University of Missouri – Kansas City.

The SAQ quantifies patients’ physical limitations caused by angina, the frequency of and recent changes in their symptoms, their satisfaction with treatment, and the degree to which they perceive their disease to affect their quality of life. Each scale is transformed to a score of 0 to 100, where higher scores indicate better function (e.g. less physical limitation, less angina, and better quality of life).

#### Minnesota Living with Heart Failure Questionnaire

The Minnesota Living with Heart Failure Questionnaire (MLHFQ) is one of the most widely used health-related quality of life questionnaires for patients with heart failure (HF). It provides scores for two dimensions, physical and emotional, and a total score. However, there are some concerns about its factor structure and alternatives have been proposed, some including a third factor representing a social dimension.

### Plans to promote participant retention and complete follow-up {18b}

Upon enrollment and randomization, the investigators will make every reasonable effort to follow the study subject throughout the entire study period. The subject is free to withdraw its written informed consent at any time. The reason for withdrawal should, if available, be documented in the subject’s medical file and in the CRF. When a subject withdraws or is withdrawn from the clinical investigation, a final clinical evaluation should be performed, documented in the medical record and in the CRF.

Participant retention will be increased by schedule strategies, e.g. including the patients in the high-risk cardiac surgery outpatient program follow-up procedure. Participants will also be reminded of the study via telephone calls from the research team.

### Data management {19}

Data entry from paper-based CRF into the statistical software SPSS will be processed by the study team. Validation of data is made by programmed checks of range, validity, and consistency. If necessary, queries are made by the study software or an authorized person. Based on the queries the investigator can check and clarify discrepancies.

After the record of all entries and clarification of all queries, the data base will be closed at the completion of the study. This performance must be documented.

### Confidentiality {27}

All local legal requirements regarding data protection will be adhered to. All study findings and documents will be regarded as confidential. The Investigator and members of the research team must not disclose any information without prior written approval from the Sponsor.

The pseudonymity of patients participating must be maintained. Throughout documentation and evaluation, the patients will be recognized on CRFs and other documents by age and identification number. Documents that identify the patient personally (e.g. the signed informed consent) must be maintained in confidence by the Investigator. The patients will be told that all study findings will be stored on a computer and handled in the strictest confidence.

### Plans for collection, laboratory evaluation and storage of biological specimens for genetic or molecular analysis in this trial/future use {33}

#### Biosamples

At the scheduled visit, blood specimens and urine samples should be collected, whereas fasting is not required. The specimens will be forwarded to the cardiosurgical research laboratory in a pseudonymous manner, which will perform the analysis.

Data arising from clinical genotyping will be subject to the confidentiality standards.

Blood and urine samples will be taken during screening, 4–12h post-surgery, 48–52h post-surgery, day 90 and day 360.

#### Left ventricular biopsies and transcriptomics/epigenomics

Intraoperative left-ventricular biopsies are performed during reperfusion phase 15 min after shock wave therapy. Two samples per treated area are collected using a 14-gauge coaxial needle. For this purpose, a standardized semi-automated biopsy system is used (Semi-Automatic Biopsy System, TSK Lab, The Netherlands) to guarantee reproducibility between patients as described previously (Treibel TA, et al. 2016, Zile MR, et al. 2015). Obtained samples are immediately washed in ice-cold saline (0.9% NaCl) and transferred in liquid nitrogen as described elsewhere (Meder B et al. 2017). Samples are transported on liquid nitrogen to the cardiosurgical laboratory, where isolation of RNA and DNA is performed. RNA and samples are thereafter subjected to screening analysis including deep sequencing, while DNA samples are analysed for high-density epigenome-wide mapping of DNAmethylation (IMGM Laboratories, Munich, Germany). Subsequent bioinformatic processing is performed in cooperation with Priv.-Doz. Dr. Hubert Hackl (Institute of Bioinformatics, MUI).

## Statistical methods

### Statistical methods for primary and secondary outcomes {20a}

#### Efficacy analysis

The primary efficacy variable is the LVEF absolute change from baseline, i.e. the intra-patient difference or change between the pre-treatment (baseline) value day 0 and the value measured at day 360. This variable is quantitative and continuous and will be presented as a mean value with 95% confidence intervals. Change in mean (μ) LVEF from baseline to day 180, defined as delta ΔLVEF, will be analysed using an analysis of covariance (ANCOVA) adjusted for LVEF at baseline (as outlined in Guideline on adjustment for baseline covariates in clinical trials EMA/CHMP/295050/2013, 5.6 ‘change from baseline’ analyses) The main study hypotheses will thus be formulated as:

$$\begin{array}{c}\mathrm H0:\;\mathrm\mu\;\;\mathrm{{\triangle}LVEFcontrol}\;=\;\mathrm\mu\;\;\mathrm{{\triangle}LVEFshockwave}\\\mathrm H1:\;\mathrm\mu\;\;\mathrm{{\triangle}LVEFcontrol}\;\neq\;\mathrm\mu\;\;\mathrm{{\triangle}LVEFshockwave}\end{array}$$

In case of severe deviations from a normal distribution, the change in LVEF from baseline to day 360 will be analysed with the non-parametric Mann-Whitney U test.

Additionally, ANOVA for repeated measurements will be applied in order to analyse LVEF changes across the full study period (day 0, day 180, day360).

#### Safety and secondary analyses

Secondary and safety parameters will be summarized using descriptive statistics, i.e. number (%) of patients for categorical variables and mean, SD (standard deviation), median, minimum/maximum for continuous variables. Descriptive statistics will be produced by the treatment group. No formal hypothesis testing will be performed. Appropriate statistical tests will be applied in an explorative manner only. Occurrence of adverse events (safety) will be compared between treatment groups using chi-square tests and Fisher`s exact tests, as appropriate. Analyses of secondary endpoints will be analysed with appropriate statistical tests such as ANOVA for repeated measurements, paired and unpaired *t*-tests as well as their non-parametric equivalents.

### Interim analyses {21b}

Two main analyses are planned: (a) An interim analysis when at least 20 participants per group had finished their 360-day follow-up for the primary endpoints, to assess the safety and to allow for an early halt to the recruiting process. (b) A final analysis after 80 patients. For both analyses, the significance level alpha will be set to 0.0294 according to the alpha spending plan proposed by Pocock.

### Methods for additional analyses (e.g. subgroup analyses) {20b}

No subgroup analyses have been planned for this trial. To investigate the robustness of the primary endpoint analysis performed in the intention-to-treat (ITT) population and to examine the possible effects of missing data, sensitivity analyses are planned. These analyses will be performed in the ITT population without the imputation of missing values, in a population with complete data sets only, and in an ITT population where the LVEF of deceased patients is set to 0%.

### Methods in analysis to handle protocol non-adherence and any statistical methods to handle missing data {20c}

The following populations will be used for statistical analysis:

#### Intention-to-treat population

The intention-to-treat population will consist of all patients who were enrolled to the study and who have given their informed consent. In case of withdrawals and missing data, the Last observation carried forward (LOCF) procedure will be applied where appropriate. The ITT population serves as the primary analysis population.

#### Modified intention-to-treat population

The modified intention-to-treat population will consist of all patients who were enrolled to the study and were clinically evaluable (per cardiac MRI).

#### Per-protocol population

The per-protocol population will include all patients who received shockwave or control treatment without major protocol deviations. Major protocol deviations will be determined and documented prior to database lock.

### Plans to give access to the full protocol, participant level-data and statistical code {31c}

The full protocol of the study will be published together with a manuscript on the results of the clinical trial. The datasets analysed during the current study and the statistical code will be available from the corresponding author on reasonable request.

## Oversight and monitoring

### Composition of the coordinating centre and trial steering committee {5d}

As this is a single-centre trial the responsibilities of a steering committee will be covered by the principal investigator and the team of co-investigators. The Steering Committee will concentrate on the progress of the study, adherence to the protocol, patient safety and consideration of new information relevant to the research question. The trial quality will be independently monitored by the Competence Centre for Clinical Trials at the Medical University of Innsbruck. The study monitor will check 100% of trial data files at least once every 6 months.

### Composition of the data monitoring committee, its role and reporting structure {21a}

#### Data Safety Monitoring Board (DSMB)

An independent committee is established by the sponsor to assess at intervals, the progress of the clinical investigation, the safety data or the critical performance endpoints and to recommend the sponsor whether to continue, suspend, modify, or stop the clinical investigation.

The DSMB will be responsible for independently evaluating the safety of the patients participating in the trial. All captured adverse events and safety reports will be reported to the DSMB for assessment. All SAEs will be forwarded to the DSMB immediately after knowledge of it. The DSMB can, if required, amend the protocol and in case the risk to the patients outweighs the potential benefit, end the study prematurely. The members of the DSBM will consist of a cardiac surgeon and a cardiologist.

#### Adverse event reporting and harms {22}

An adverse event is the appearance or worsening of any undesirable sign, symptom, or medical condition occurring during or after the treatment (DESWT + CABG). Medical conditions/ diseases present before the treatment will only be considered adverse events if they worsen after the treatment. Abnormal laboratory values or test results constitute adverse events only if they induce clinical signs or symptoms, are considered clinically significant, or require therapy. The occurrence of adverse events should be sought (1) direct contact to the treating physicians, (2) screening of medical records and laboratory data for anticipated adverse events listed in the CRF and (3) by non-directive questioning of the patient at each visit during the study. Adverse events also may be detected when they are volunteered by the patient during or between visits or through physical examination, laboratory test, or other assessments.

#### Frequency and plans for auditing trial conduct {23}

Monitoring and audits are performed for the quality assurance within the clinical investigation.

Monitoring and auditing procedures developed or endorsed by the Sponsor will be adhered to, in order to comply with EN ISO 14155 guidelines and local legal requirements to ensure the acceptability of the study data.

Pre-investigational visits will be done to ensure the suitability of each participating centre. Monitoring visits by representatives of the Sponsor will be carried out to review study plan compliance, to compare CRFs and individual patient’s medical records, to perform accounting of study material, and to ensure that the study is being conducted according to pertinent regulatory requirements. CRF entries will be verified with source documentation. The frequency and duration of Monitoring visits will be determined according to clinical site accrual, site performance, adherence to the protocol, and data quality.

#### Plans for communicating important protocol amendments to relevant parties (e.g. trial participants, ethical committees) {25}

After the protocol has been submitted to the ethics committee (EC), any substantial change will require a formal amendment. The amendment must be signed by all of the signatories to the original protocol. Once the study has started, amendments should be made only in exceptional cases. The ethics committees must be informed of all amendments. Approval must be sought for ethical aspects and must also be obtained from the competent authorities.

#### Dissemination plans {31a}

Key trial results will be presented in national and international conferences. There are no publication restrictions. The trial results will be made accessible to the public in scientific journals. For all publications, the data protection of the subjects will be maintained. The study data are the property of the Medical University of Innsbruck. The data from the whole trial can be published separately.

## Discussion

Ischemic cardiomyopathy due to coronary artery disease remains a major burden for affected patients and, thus, represents a major challenge for Western health care systems. Post-infarctional remodelling and replacement of contractile myocardium with dysfunctional scar tissue lead to alteration of left-ventricular geometry and, hence, cardiac output for organ perfusion resulting in heart failure. Congestion, fatigue, dyspnea and angina severely impact on patients’ quality of life and can cause repeated episodes of cardiac decompensation concomitant with the need for hospital admission. Patients with heart failure due to ischemic cardiomyopathy have a decreased life expectancy [12]. Current neurohumoral treatment strategies mainly aim at the improvement of symptoms. Complete revascularization of viable myocardium remains a cornerstone in the treatment of ischemic cardiomyopathy. The available scientific evidence currently favours CABG over PCI in patients with multivessel disease and impaired LV systolic function [13]. However, CABG surgery represents a somewhat palliative strategy, as it mainly aims at avoiding novel MI rather than regenerating myocardium and improving contractility. SWT represents a promising therapeutic tool for the regeneration of dysfunctional tissue. It has proven effective in numerous pathologies, mainly by induction of neovascularization and modulation of inflammation. Extensive preclinical studies in small animals as well as large animals show a clear benefit of SWT for the functional restoration

of ischemic myocardium. For this purpose, the CAST-HF trial was initiated in 2018, representing the first randomized controlled trial to evaluate the benefit of direct cardiac shockwave therapy. Based on well-described evidence from in-vitro as well as numerous small and large animal experiments, this trial marks the next milestone to develop direct cardiac SWT for broad clinical routine use in patients suffering from ischemic heart failure [10, 14–19]. Thus, it could become the first available treatment option for the regeneration of ischemic myocardium. In contrast to other experimental treatment strategies, SWT has been used for many decades in medicine, and to date, there are no unfavourable long-term side effects reported. In contrast, other promising experimental approaches for myocardial regeneration could not be translated into a clinical setting. Stem cells for myocardial regeneration have been investigated intensively. Despite promising preclinical results, the clinical translation has not been successful so far due to reports of arrhythmogenic events, lack of efficacy, and failed incorporation into the site of injury.

### Trial status

Date: 11.11.2022

Protocol version: 1.4

Effective Date: 13.10.2021

First Patient First Visit (FPFV): 28.11.2018

Trial status: Active, not recruiting

Recruitment closed on 01.06.2022

Last Patient Last Visit (LPLV) is expected in April 2023.

## Abbreviations

CABG: Coronary-artery bypass-graft surgery
CAD: Coronary arterial disease
CAST-HF: Direct cardiac shockwave therapy in patients undergoing coronary artery bypass grafting
CK-MB: Creatine kinase
CRP: C-reactive protein
CSP: Cardiac Shockwave Probe
DAMPS: Dangerassociated molecular patterns
DESWT: Direct epicardial shockwave therapy
DSMB: Data Safety Monitoring Board
EF: Ejection fraction
EV: Extracellular vesicles
FGF: Fibroblast growth factor
GFR: Glomerular filtration rate
LVEF: Left-ventricular ejection fraction
NYHA: New York Heart Association
MI: Myocardial infarction
MLHFQ: Minnesota Living with Heart Failure Questionnaire
MRI: Magnet resonance imaging
PCI: Percutaneous coronary intervention
PlGF: Placental growth factor
SAQ: Seattle Angina Pectoris Questionnaire
SF36: 36-item short-form survey
SDF-1: Stromal-cell-derived factor 1
SWT: Shockwave therapy
TLR3: Toll-like receptor 3
TropT: Troponin T
VEGF: Vascular endothelial growth factor
VEGFR2: VEGF receptor 2
